# An Example of the Concurrent Development of Cancer and Tubercle

**Published:** 1854-09

**Authors:** Septimus Wm. Sibley

**Affiliations:** Registrar to the Middlesex Hospital


From the Lancet

### *An Example of the Concurrent Development of Cancer and Tubercle.* By Septimus Wm. Sibley, Registrar to the Middlesex Hospital.

(*Communicated by* Mr Arnott.)

This was the case of a woman, aged forty-eight, admitted into the Middlesex Hospital, with a sloughing cancerous sore in the left breast; there was a hard tumour on the inner side of the size of an orange, and several small nodules of cancer at its edges. In the course of five days after her admission nearly the whole remaining portion of the tumour sloughed away, leaving a clean looking surface, which immediately began to cicatrize. Subsequently, pulmonary symptoms became developed, profuse expectoration followed, and she sank and died three months after her admission.—On making a section of the structure of the left breast, it was seen to be an extremely dense form of infiltrating scirrhus, traces of breast tissue, such as ducts, being very apparent. In the thorax, large masses of tuberculous lung tissue were observed. Tubercular cavities existed in the apices of both lungs; a part of the lower lobe of the right lung was in a state of green hepatization, and the bronchial tubes were thickened and dilated. In the left pleura were numerous crude tubercles. On examining the dates of this case, positive proof was obtained that a cancerous tumour was increasing in the breast simultaneously with the increase of tubercular disease of the lungs and that for a period of at least six weeks. The author thought that a single instance of the concurrent existence of these diseases was sufficient to destroy the doctrine of the absolute incompatibility of tubercle and cancer with each other. The paper concluded with some appropriate remarks on the constitutional diathesis tending to the concurrent development of these two diseases.

